# Subverting *Attachment* to Prevent *Attacking*: Alteration of Effector Immune Cell Migration and Adhesion as a Key Mechanism of Tumor Immune Evasion

**DOI:** 10.3390/biology13110860

**Published:** 2024-10-24

**Authors:** Marta Mastrogiovanni, Emmanuel Donnadieu, Rajiv Pathak, Vincenzo Di Bartolo

**Affiliations:** 1 Department of Developmental and Molecular Biology, Albert Einstein College of Medicine, Bronx, NY 10461, USA; 2 Gottesman Institute for Stem Cell Biology and Regenerative Medicine, Albert Einstein College of Medicine, Bronx, NY 10461, USA; 3 Equipe Labellisée Ligue Contre le Cancer, CNRS, INSERM, Institut Cochin, Université Paris Cité, F-75014 Paris, France; emmanuel.donnadieu@inserm.fr; 4 Department of Genetics, Albert Einstein College of Medicine, Bronx, NY 10461, USA; rajivpathak17@gmail.com; 5 Immunoregulation Unit, Institut Pasteur, Université Paris Cité, F-75015 Paris, France; vincenzo.di-bartolo@pasteur.fr

**Keywords:** effector immune cells, immune cell biology, leukocyte adhesion, leukocyte migration, tumor microenvironment

## Abstract

Cell adhesion regulates specific migratory patterns, location, communication with other cells, physical interactions with the extracellular matrix, and the establishment of effector programs. Proper immune control of cancer strongly depends on all these events occurring in a highly accurate spatiotemporal sequence. In response to cancer-associated inflammatory signals, effector immune cells navigating the bloodstream shift from their patrolling exploratory migration mode to establish adhesive interactions with vascular endothelial cells. This interaction enables them to extravasate through the blood vessel walls and access the cancer site. Further adhesive interactions within the tumor microenvironment (TME) are crucial for coordinating their distribution in situ and for mounting an effective anti-tumor immune response. In this review, we examine how alterations of adhesion cues in the tumor context favor tumor escape by affecting effector immune cell infiltration and trafficking within the TME. We discuss the mechanisms by which tumors directly modulate immune cell adhesion and migration patterns to affect anti-tumor immunity and favor tumor evasion. We also explore indirect immune escape mechanisms that involve modifications of TME characteristics, such as vascularization, immunogenicity, and structural topography. Finally, we highlight the significance of these aspects in designing more effective drug treatments and cellular immunotherapies.

## Simple Summary

The role of our immune system is to protect us from multiple pathogens as well as cancer cells. To fulfill this task, immune cells, or leukocytes, circulate within our blood to detect and respond to signs of infection, inflammation, or other abnormalities throughout the body. When they encounter these signals, they cease their patrolling and exploratory blood navigation to establish productive interactions with the vasculature. In this way, they can emigrate from the blood and access specific tissues, such as solid tumors. Once infiltrating into the tumor area, effector immune cells, such as T cells and NKs, must migrate to contact malignant cells and kill them. Upon activation, these cells help recruit additional immune cells, enhancing the overall anti-tumor immune response. These migratory and interactive steps all depend on the expression of specific molecules that enable immune cells to sense and respond to environmental signals and regulate their adhesion and interactions with other cells. Nonetheless, tumors have developed several strategies to evade immune detection and hinder immune control. In this review, we explore the mechanisms tumors use to limit effector leukocyte infiltration and suppress anti-tumor immunity. Furthermore, we discuss how this knowledge can inform the development of more effective cancer immunotherapies.

## 1. Introduction

The immune system’s ability to recognize and eliminate malignant cells is essential for maintaining tissue homeostasis and preventing cancer development. Key anti-tumor cellular players are effector immune cells, such as cytotoxic CD8+ T lymphocytes (CTLs) and natural killer (NK) cells. Furthermore, a critical component of this immune surveillance is the precise regulation of their migration and adhesion, processes that are controlled by interactions between immune cells, the extracellular matrix (ECM), and stromal and vascular structures [1]. These interactions not only facilitate the migration of effector leukocytes to sites of potential danger but also their subsequent activation and effector functions within tissues, including the tumor microenvironment (TME).

Cell adhesion is necessary for a wide range of vital processes, including embryogenesis, angiogenesis, tissue renewal and repair, and immune responses. Immune cells rely on adhesion to distribute within lymph nodes or tissues, such as solid tumors, and establish contact with antigen-presenting cells and infected or malignant cells. The modalities and features of cell adhesion depend on the surface expression and orchestrated action of chemokine and adhesion receptors, which dictate specific migration modes, regulating the switch from an exploratory to an infiltrating phenotype. Furthermore, these receptors mediate physical interactions with the ECM and communication with other cells, serving recruitment purposes and/or guidance instructions. Proper regulation of adhesion-dependent mechanisms by chemokines and adhesion receptors is also essential for the establishment and execution of effector functions.

Building on this understanding of cell adhesion, it becomes evident that these processes are integral to immune cell function, particularly in the context of tumor immunity. Effector immune cells such as NK cells and CD8+ T cells play a vital role in tumor eradication. High densities of anti-tumor immune cell populations, such as cytotoxic T cells, are key indicators of favorable prognosis in many cancers [2,3]. The mobilization of these cells from the blood vasculature and emigration into tumors require tight coordination between adhesive and migratory events. Likewise, once within the TME, both infiltrating and tissue-resident leukocytes have to adopt specific migratory patterns, helping them to redistribute into various specialized niches. This redistribution allows them to functionally interact with each other, with stroma cells, and, ultimately, with malignant cells. Notably, all stages of the anti-tumor immune response depend on adhesion and migratory cues. Therefore, the proper orchestration of these adhesion features profoundly impacts the functionality and behavior of immune cells, shaping the tumor outcome. The goal of this review is to illuminate the importance of adhesive cues in the regulation of key anti-tumor immune cell functions, such as TME infiltration and interaction with malignant cells. In particular, we will discuss how subversion of these adhesion-dependent functions can hamper immune surveillance, thus favoring tumor escape.

The TME is a complex agglomerate composed of tumor cells, stromal tissue, and the surrounding ECM. The stromal tissue includes both immune and non-immune cells, for example, fibroblasts and endothelial cells of the blood vasculature, which support tumor growth. In this review, we will report the negative impact of the cellular and non-cellular components of the TME on immune cell surveillance. Moreover, we will review those active mechanisms adopted by tumors to facilitate their escape from immune control, which involve the modulation of immune cell adhesion and migration cues. For example, we will review the mechanisms adopted by tumors to alter the expression of chemokine and adhesion receptors on the surface of immune cells, impairing their migratory ability, extravasation, or distribution within specialized sub-regions of the tumor niche. Additionally, we will explore how modifications of the TME, including alterations in vascularization, immunogenicity, and structural topography, contribute to immune evasion. Our final goal is to shed light on the biological aspects of immune cell adhesion and migration in the context of immune surveillance. Specifically, we will provide a few examples of how the assessment of these features can enhance the efficacy of some immune-centered anti-cancer strategies, such as CAR-T therapy.

## 2. Overview of the Molecular Players Involved in Immune Cell Adhesion and Migration into and Within Solid Tumors

### 2.1. Chemokine Signaling Guides Immune Cell Recruitment in the Tumor

Chemokine receptors are 7 transmembrane G protein-coupled receptors (GPCRs) predominantly expressed on the surface of leukocytes. These receptors bind to four corresponding subfamilies (named C, CC, CXC, and CX3C) of chemotactic chemokines that modulate immune cell trafficking and migration patterns both under homeostatic conditions and during inflammatory or adaptive immune responses [4]. “Homeostatic” or “constitutive” chemokines are constitutively secreted in discrete sites within lymph nodes, skin, and mucosa and are essential for maintaining physiological traffic and positioning of cells. In contrast, resident and infiltrating cells within inflamed tissues, such as tumors, secrete “inflammatory” or “inducible” chemokines to recruit effector cells.

Chemokine axes play several physiological roles. They regulate immune cell migration dynamics and guide tissue distribution and trafficking. They orchestrate the steps of the “adhesion cascade”, including the firm arrest of leukocytes on the blood vessel wall and diapedesis, which enables their infiltration into the underlying tissues. Additionally, chemokine signaling is also crucial for favoring antigen cross-presentation, T cell priming, and T cell activation. Chemokines act in a soluble manner but also in interactions with glycosaminoglycans, particularly heparan sulfate, in the extracellular matrix. This interaction immobilizes chemokines, creating stable local gradients that coordinate cell movement and adhesion [5].

#### Chemokine Signaling in the Tumor Guides Immune Cell Recruitment and Orchestrates Immune Cell-APC Interactions

Within tumors, chemokines modulate the phenotype and functions of both cancer cells and the surrounding microenvironment, including non-cellular components. Indeed, it is challenging to define the specific role of each chemokine in the tumor context and to determine whether a particular chemokine axis is beneficial or detrimental. Therefore, targeting the chemokine system therapeutically presents a significant challenge for the biomedical field [6,7].

**Figure 1:**
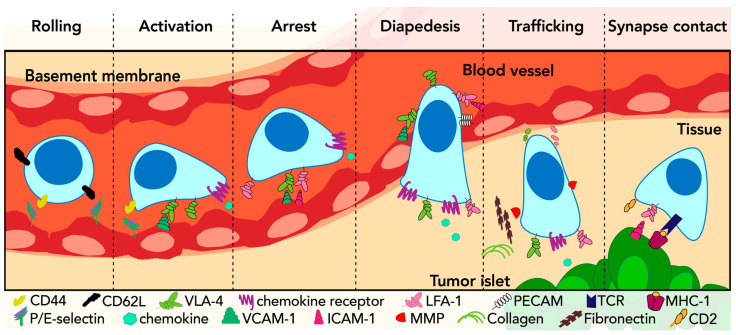
Adhesion cues mediate all the steps of immune cell infiltration into tumors. Selectins and their ligands mediate immune cell rolling on the surface of vascular endothelial cells. Chemokine-dependent activation of adhesion receptors, such as VLA-4 and LFA-1 integrins, leads to their conformational change and firm arrest of leukocytes on the vasculature wall. Further integrin-mediated adhesion cues facilitate cytoskeleton rearrangements necessary for leukocyte transmigration towards the underlying tissue. Integrins also mediate immune cell crosstalk with both components of the ECM and other cells, influencing both the trafficking within the tumor tissue and the communication with the surrounding niche. Finally, the strength and duration of LFA-1/ICAM-1-dependent contact between immune effector cells and target cancer cells will determine the efficacy of the anti-tumor immune cell activity. Adapted from the model of leukocyte trafficking through the blood and its adhesion to the vessel wall proposed by E. C. Butcher in 1991 [11].

Chemokine signaling pathways are utilized by both immune cells to migrate into the tumor microenvironment (Figure 1) and by cancer cells to emigrate and invade new locations. Several studies have provided evidence of the association of the up- or down-regulation of chemokines and their receptors with the degree of immune cell infiltration and distribution within the tumor [7,8,9,10]. For example, CXCR6-driven signaling in CTLs has been linked to the optimal positioning of these cells within specific tumor areas enriched with CXCL16-expressing dendritic cells [10].

The expression of CXCR3 receptor ligands has been associated with T-cell infiltration in melanoma and NK cell accumulation in lymphoma and melanoma [8,9]. However, CXCR3 is also expressed in immunosuppressive regulatory T cells (Tregs) that are particularly enriched in several human cancers, including ovarian and liver carcinomas [12,13]. Recent evidence has shown that CXCR3 is critical for Treg cell suppression of antitumor CD8+ T cells due to the preferential interaction with dendritic cells (DCs) [14]. Furthermore, disrupting Treg’s CXCR3 boosted tumor CD8+ T cells and slowed cancer progression in solid tumor mouse models [14]. These data highlight CXCR3 as a key receptor whose expression in specific immune cell types, immunosuppressive or effector, can regulate both the type and intensity of the anti-tumor immune response, shaping the tumor outcome and potentially determining the efficacy of therapeutic interventions. Hence, CXCR3’s role in tumor immunology warrants further investigation.

The expression of specific chemokines and chemokine receptors has also been associated with the efficacy of interactions between immune cells and antigen-presenting cells (APCs) within tumors. For example, increased expression of CXCL9 by tumor-associated DCs has been shown to enhance their interaction with CXCR3-expressing CD8+ T cells in a murine model of breast cancer [15]. Conversely, CXCL9 and CXCL10 produced by DCs have been shown to increase the contact with CXCR3+ CTLs, thereby enhancing the efficacy of programmed cell death protein 1 (PD-1) blockade therapy [16,17]. Interestingly, CXCL9 and CXCL10 produced by tumor-associated macrophages (TAMs) have also been correlated with enhanced anti-programmed death-ligand 1 (PD-L1) response rates [18]. Thus, although suppressive functions are generally attributed to TAMs, this specific role of CXCL9 and CXCL10 in anti-tumor immunity has suggested a potential use of TAMs to prevent T cell exhaustion and promote anti-PD-L1 responses [19,20]. Further studies are therefore necessary for both clarifying the specific role of the different TME immune cell populations and for elucidating the mechanisms underlying the CXCL9/CXCL10-PD-L1 crosstalk.

Although the upregulation of some chemokine axes has been associated with tumor growth and poor prognosis [21,22,23,24], little is known about the active initiation of these mechanisms by tumors to ultimately favor their immune escape. In the following sections, we will review escape mechanisms employed by tumors to induce or disrupt the expression of chemokines and chemokine receptors on the surface of leukocytes or surrounding cells, thereby reducing immune surveillance.

### 2.2. Cell Adhesion Molecules Shape Immune Cell Migration Modes, Interactions, and Effector Functions

#### 2.2.1. Cell Adhesion Molecules in the Immune System

Cell adhesion molecules (CAMs) include selectins, integrins, cadherins, and the immunoglobulin superfamily of cell adhesion molecules (IgCAMs). The role of these membrane proteins is to establish adhesive interactions with both the extracellular matrix and neighboring cells in order to modulate migration modes, cellular crosstalk, and functions.

- Selectins

Selectins are a family of glycoproteins containing a C-type lectin motif that binds specific carbohydrate structures in a calcium-dependent manner. The selectin family includes three subsets, reflecting specific tissue distribution: E-selectin (CD62E) is expressed on endothelial cells, L-selectin (CD62L) on leukocytes, and P-selectin on platelets and endothelial cells.

Selectins mediate short-lived (i.e., lasting less than one second) and reversible interactions among cells [25]. For example, CD62L expressed on circulating leukocytes interacts with P-selectin glycoprotein ligand-1 (PSGL-1) and other glycosylated ligands on the surface of vascular endothelial cells, allowing immune cells to slow their bloodstream navigation and tether and roll along the vascular wall (Figure 1). Moreover, the concomitant expression of CD62L and PSGL-1 on the surface of the same leukocyte facilitates their reciprocal interactions, further promoting tethering.

- ICAM-1, VCAM-1, and H-CAM

The transmembrane glycoprotein receptor intercellular adhesion molecule 1 (ICAM-1) is expressed at low levels in multiple cell types, including endothelial cells, immune cells, and tumor cells. However, under certain cytokine stimuli (e.g., inflammation-induced IFNγ, IL-1, or TNF-α secretion), its expression increases, enhancing adhesion between endothelial cells and immune cells. ICAM-1 regulates intercellular adhesion and communication and is involved in many essential cellular responses to inflammation or injury [26]. Moreover, it is a key regulator in the interactions between immune effectors and tumor cells, with significant implications for antitumor immunity and therapeutic approaches [27,28].

Herzfeldt et al. have highlighted the essential role of membrane-bound ICAM-1, rather than soluble ICAM-1, in mediating tumor-cell killing [29]. On the other hand, soluble ICAM-1 released by tumor cells inhibited NK functions in patients with colonic carcinoma [30], thus suggesting a mechanism of tumor immune escape. Recent work by Alon and colleagues has demonstrated that during viral infections, ICAM-1 is necessary for strengthening the DC-T cell interactions and is dispensable, in turn necessary for T cell differentiation in Th1 [31]. These findings reveal that the common assumption of sustained, long-lasting ICAM-1-dependent DC-T cell contacts dictating the differentiation of the latter into CTLs might be revised. Such new insights should inspire further dissection of these ICAM-1-dependent interactions in the tumor context.

VCAM-1 (vascular cell adhesion molecule 1) is also expressed by several cell types, particularly those involved in immune and inflammatory responses. Although predominantly expressed by endothelial cells, during acute inflammation or chronic conditions in some diseases, VCAM-1 can be expressed by non-endothelial cells (e.g., cancer cells, macrophages, DCs) [32]. Pro-inflammatory cytokines (e.g., TNFα) and reactive oxygen species (ROS) can activate the expression of VCAM-1 [33]. Several studies have correlated VCAM-1 expression and function with tumor angiogenesis. Indeed, serum VCAM-1 levels have been associated with the microvessel density in breast cancer [34], and angiogenic signals have been described to stimulate VCAM-1 gene expression in endothelial cells [35].

Furthermore, VCAM-1 is involved in tumor metastasis, as it has been described in lung and colorectal cancers [32,36,37]. Interestingly, imaging assessment of VCAM-1 density before treatment initiation has resulted in information about the T-cell infiltration within the tumor vasculature and about the response to PD-L1 blockade in preclinical models of colorectal cancer [38]. In fact, T-cell-mediated tumor rejection is inhibited by blocking T-cell binding to either VCAM-1 or ICAM-1 on endothelial cells. This highlights the key role of adhesion molecules for T-cell infiltration and the relevance of infiltrating T cells in tumor eradication [38].

CD44 is a type I transmembrane glycoprotein with a C-type lectin motif that binds hyaluronic acid in the ECM and E-selectins on activated endothelial cells. CD44 upregulation is typically associated with T-cell activation and memory. However, CD44 has also been described as important for the initial steps of rolling that precede VLA-4-VCAM-1 interactions [39]. Accumulating evidence indicates that CD44 is involved in various cancer-associated pathways, and, in recent years, there has been an increasing awareness of the plethora of functions of CD44 in the tumor context. Xu et al. provide a detailed review of CD44 in different tumors and upon interactions with different cancer ligands [40].

- Integrins

Integrins represent the main class of adhesion molecules responsible for the establishment of adhesive interactions with both the extracellular matrix and neighboring cells. Integrins are heterodimers composed of various α and β subunit combinations and can exist in three ligand affinity states—low, intermediate, and high—depending on their activation state. Integrin function depends on two mutually cooperative pathways: inside-out signaling by other receptors, such as chemokine receptors, and outside-in signaling triggered by the binding of the integrins themselves to multivalent ligands [41,42]. Integrin activation leads to integrin clustering and assembly of the focal adhesion complex, which is a large cytoplasmic signaling platform including cytoskeleton players and force transducers, such as talin [41,42]. Evidence has pointed to ion channels as interactors of integrin receptors since integrin engagement has been demonstrated to activate ion flux [43,44]. Indeed, integrin-mediated cell–ECM and cell–cell adhesion events involve ion transport [42,44,45].

Very-late antigen 4 or VLA-4 (α4β1 or CD49d/CD29 integrin) and lymphocyte function-associated antigen-1 or LFA-1 (αLβ2 or CD11a/CD18 integrin) are two critical integrins involved in the leukocyte adhesion cascade. They participate in the rolling and tethering steps; however, their affinity increases upon chemokines-induced activation [46] (Figure 1). Consequently, VLA-4 and LFA-1 enhance adhesion to their respective ligands, VCAM-1 and ICAM-1, which are expressed on the surface of endothelial cells (Figure 1). VLA-4 and LFA-1 mediate the firm arrest of leukocytes to the vasculature wall, ultimately arresting their movement and initiating the trans-endothelial migration, also known as diapedesis or extravasation [47]. During diapedesis, immune cells encounter three distinct barriers: the endothelial cells of the vasculature, the endothelial cell basement membrane, and pericytes, which are perivascular cells forming a wall around blood capillaries. During this process, leukocytes can engage endothelial cell junctional adhesion molecules, such as the junctional adhesion molecule JAM-A, through LFA-1 [48]. LFA-1 is also crucial for synapse formation with APCs for antigen presentation and for the process of T cell-mediated cytotoxicity. Thus, effective T-cell interactions with target malignant cells rely on the binding between LFA-1 and ICAM-1, expressed on their respective membrane surfaces [49,50]. In particular, the nanocluster organization of LFA-1 allows for the calibration of T-cell killing in response to antigen stimulation strength [51]. Of note, leukocyte interaction with tissues expressing both VCAM-1 and ICAM-1 results in a “trans-regulation” between VLA-4 and LFA-4 integrins. Such a mechanism may differentially affect various cell types (e.g., lymphocytes vs. myeloid cells), thus potentially modifying their relative abundance in tumor infiltrates [52].

LFA-3, also known as CD58, is an adhesion molecule broadly distributed in human hematopoietic and non-hematopoietic cells. Its ligand, CD2, is primarily expressed on the surface of T cells and NKs and is required for promoting cell adhesion and recognition preceding immunological synapse formation and T-cell receptor (TCR) signaling [53,54]. CD2 has been described to be enriched on the microvilli of human effector T cells, facilitating the initial antigen recognition events and therefore sensitizing and potentiating the TCR signaling [55]. Indeed, CD2-enriched microvilli at the IS have been considered key signaling hubs or “immunological synaptosomes”, necessary for facilitating the detection of and increasing the sensitivity to cognate antigens [55,56] (Figure 1).

Finally, CD103 (αEβ7 integrin) is predominantly expressed by intraepithelial T lymphocytes and CD8+ tumor-infiltrating lymphocytes (TILs) and mediates their adhesion and tissue retention by binding E-cadherin, which is abundant on epithelial cells [57].

#### 2.2.2. Adhesion Cues Mediate the Establishment of Productive Immune Cell-to-Cell Contacts

While chemokine signaling directs immune cell infiltration into the tumor microenvironment by providing instructive cues, adhesion shapes this process by participating in the mechanical interpretation of these guidance signals. Therefore, the entry of immune cells into tumor tissue implies interactions with the endothelial cells of the vasculature. Similarly, the cytotoxic activity of immune cells against malignant cells relies on a close interplay between these cell types. These interactions are not random but are critically regulated by adhesive cues. Nonetheless, the nature of these interactions ultimately determines the outcome of anti-tumor immune responses.

- The adhesion cascade

Leukocyte recruitment and trafficking within a tissue require adhesion-dependent events enabling their migration through blood vessel walls [47]. The three main steps of the adhesion cascade originally proposed by E.C. Butcher in 1991 [11] are schematically summarized in Figure 1. These steps necessitate an active coordination among diverse and specific adhesion molecules and chemokine receptors. As such, the adhesion cascade represents the most accurate regulatory process governing cell adhesion and migration dynamics, allowing immune cells to undergo emigration from the bloodstream into underlying tissues [58].

Initially, selectins are engaged and mediate leukocyte deceleration, facilitating their rolling on the surface of vascular endothelial cells. Subsequently, chemokine signaling is necessary for cell activation, which stimulates the surface exposure of further chemokine receptors and adhesion molecules. These molecules, in turn, stabilize the adhesions to the vasculature, leading to the firm arrest of the cells from their navigation in the bloodstream. This is a crucial step preceding diapedesis, whereby immune cells move from the vasculature into the underlying tissues [47].

- Immunological synapses

Immunological synapses (ISs) are specialized cell-to-cell contacts established between immune cells, such as T cells, and APCs, or target cells. These interactions lead to the priming of T cells (“stimulatory ISs”) or trigger T cell effector functions, such as cytotoxic activity (“effector ISs”). Both types of interactions involve adhesive cues and take place in the tumor microenvironment. Thus, characterization of the adhesive molecules mediating IS formation and function is key for better understanding and manipulating the local anti-tumor immune activity and possibly enhancing current cancer immunotherapy.

IS formation between T cells and APCs, or tumor cells, is a highly dynamic process. During the initial contacts, integrins mediate the engagement of the cell surfaces, and they are distributed throughout the cell–cell interface or more abundant in the center. At later time points, the TCR interaction with the major histocompatibility complex (MHC)/peptide complex triggers cell activation, and adhesion molecules may cluster in different, juxtaposed areas of the synapse as compared to the TCR and signaling molecules [59]. This segregation in distinct “supramolecular clusters” may assume different patterns depending on the type of APC or target cell, the maturation state of the T cell, or the amount and affinity of the stimulating antigen [60]. Such different patterning may influence the strength and stability of the interaction. While a stable engagement may not be essential for T cell activation, it is crucial for halting T cell migration and facilitating the attack on tumor cells [61,62]. Notably, both chemokine- and TCR-dependent signals are critical for enhancing LFA-1 activation and affinity for ICAM-1. This leads to increased integrin engagement with ICAM-1 and stabilization of cell–cell interactions [63].

LFA-1 plays a pivotal role in the core architecture and signaling of the IS. It is indeed necessary for stimulating the initial adhesiveness to the target cell and for modulating T-cell cytotoxic activity [49,50,51]. The maturation of cytotoxic IS and activation of effector immune cell functions depend on the involvement of LFA-1 and other adhesion molecules, such as CD103 and CD58. Together with TCR engagement, CD103- and CD58-mediated adhesive interactions have been described to enhance the strength of the T cell/target cell interaction and boost both IS signaling and effector activity [53,64]. Furthermore, CD2-enriched microvilli have been found to facilitate the surveying of antigen-presenting cells and to enhance T cell TCR activation [55,56]. Interestingly, the releasing of microvilli particles containing CD2 has been involved in activating dendritic cells, thus enhancing antigen recognition and binding [65].

Hence, a detailed understanding of how various adhesion molecules mediate all the steps of formation and function of the IS would permit therapeutic exploitation of these molecules in cancer treatment.

### 2.3. The Adhesion and Chemokine Systems Mediate Large-Spectrum Immune Cell Responses

The adhesion and chemokine systems are essential not only for mediating immune cell recruitment and trafficking but also for establishing proper contacts necessary for cellular communication and the precise execution of anti-tumor cytotoxicity. Therefore, mechanisms that facilitate in situ homotypic and heterotypic cell crosstalk sculpt the type and magnitude of anti-tumor immune responses.

#### Additive Infiltration

Immune cells within the TME can communicate and accelerate the recruitment of additional immune cells to the tumor site through the diffusion of soluble chemokines or the deposition of chemokine trails. This process, known as ‘additive infiltration’, can ultimately improve anti-tumor immune efficacy.

Immune cell recruitment by other leukocytes can be homotypic, as described by Galeano Niño et al. [66]. In this study, the authors demonstrated that antigen-engaged CTLs promote the rapid convergence of additional CCR5-positive CTL cells within tumor spheroids by the increased release of the CCL3 and CCL4 chemokines. Moreover, the release of migration tracks through a process termed “membrane ripping” represents another mechanism of additive migration. Briefly, immune cells can leave behind macroaggregates containing cytokines, integrin, surface receptors, and miRNAs after their passage [67,68]. In this way, following immune cells receive guidance signals leading to their coordinated recruitment and action. Likewise, during an influenza infection, neutrophils can leave behind molecular “trails” composed of chemokines, cytokines, or other molecules that can attract and guide CD8+ T cells [69].

Despite mechanisms of membrane ripping not being described for effector immune in the tumor context, these lines of evidence suggest that the alteration in adhesion and chemokine cues do not merely affect individual cells. The sabotage of adhesive signals might impair collective immune behavior and cooperation, thereby accelerating immunosuppression incrementally.

## 3. Modulation of Immune Surveillance by the Tumor Microenvironment

The TME includes tumor malignant cells that co-exist with both immune cells and other cellular and non-cellular components. Their mutual interaction leads to an environmental phenotypic and functional plasticity that co-evolves with malignant cells. The resulting complex ecosystem plays an essential role in determining the rate of recruitment, density, composition, and distribution of immune cell infiltrates, as well as their phenotype and function [70], thus defining the outcome of anti-tumor immune responses. The overall phenotype of the TME, whether “pro-tumor” or “anti-tumor”, will depend on the ability of immune infiltrates to communicate with each other and interact with non-immune and tumor cells [70].

### 3.1. Components of the TME

#### 3.1.1. TME Immune Cell Infiltrates

Immune infiltrate densities vary from tumor to tumor, between tumor types, and between primary tumors and metastases [2,71]. Moreover, immune infiltrate composition changes both temporally (tumor stages) and spatially (tumor subareas), influencing immune surveillance and thus having a major impact on tumor progression [72,73,74,75]. Tumors also undergo dynamic immunoediting, inducing temporal heterogeneity in the TME. This implies an initial phase of transformed cell elimination by innate and adaptive immune cells, a phase of equilibrium between the tumor and the immune system, and eventually leads the tumor to acquire an immune escape phenotype [75,76]. Further, the TME is characterized by immune cell spatial heterogeneity. As such, spatially confined sub-tumor microenvironments can be distinguished based either on the enrichment of the total immune infiltrate or on the enrichment of specific infiltrating immune cells over others [77,78]. For example, the stroma is often more enriched in macrophages and NK cells, whereas intratumoral spaces are more often invaded by neutrophils, and lymphoid islets or tertiary lymphoid structures (TLSs) adjacent to the tumor nests are where both DCs and T cells are often more abundant and where antigen presentation takes place [2,79,80]. This tumor profile, referred to as “immune excluded”, is characterized by cytotoxic T cells, enriched in the tumor stroma but unable to penetrate the tumor core [81]. Nevertheless, the TME “immune contexture”, i.e., “the density, phenotype, activation status, and location of immune cells” [82], in time and space, is not only crucial for dictating the cancer fate but is also clinically relevant as a prognostic factor for human cancer survival [3,82,83,84].

Immune cell infiltrates include macrophages, neutrophils, monocytes, DCs, mast cells, eosinophils, myeloid-derived suppressor cells, platelets, innate-like lymphocytes, NK cells, NKT cells, γδ T cells, and all T cell populations and B cells [85]. Herein, we briefly summarize those immune populations whose adhesion-dependent functions are better characterized in the tumor context.

- Myeloid cells

TAMs represent 50–70% of the immune landscape, resulting in the most abundant immune cell population in the TME [86]. Both infiltrating and tissue-resident macrophages co-exist in the TME in both M1-like and M2-like functional statuses. However, during the different phases of tumor progression, macrophages undergo dynamic transcriptomic and phenotypic changes, ultimately leading to the activation of pro-tumorigenic functions, more likely M2-type [87]. TAMs contribute to tumor progression by expressing PD-L1 to inactivate cytotoxic T cells and by producing various cytokines and chemokines necessary for processes such as Tregs polarization and function, as well as for shaping the extracellular matrix. TAMs more often correlate with promoting angiogenesis, tissue remodeling, and repair, thereby favoring immune escape mechanisms, tumor growth, and malignancy. Thus, their accumulation is associated with reduced prognosis in several human cancers [82,88,89].

Myeloid-derived suppressor cells (MDSCs) are immature myeloid cells that contribute to tumor growth and progression by establishing an immunosuppressive TME. Among their pro-tumor functions, MDSCs can induce the de novo generation of Tregs and the differentiation of M2-like macrophages through the secretion of TGF-β and IL-10. Additionally, MDSCs secrete ROS, which are toxic for most of the immune cells in the TME and are responsible for stimulating vasculature endothelial growth factor (VEGF)-driven angiogenesis. MDSCs can also alter the metabolite availability in the TME and express negative immune checkpoints [90]. Overall, these mechanisms mediate the immunosuppressive functions of MDSCs.

Similar to TAMs, tumor-associated neutrophils (TANs) can acquire either a pro-tumor (N2-type) or an anti-tumor (N1-type) phenotype following cytokine stimulation. The N2-type phenotype is characterized by the up-regulation of chemokines such as CCL2, CCL4, and CXCL12, while the N1-type phenotype is characterized by the up-regulation of TNFα, CCL3, and ICAM-1 [91,92]. Despite being poorly characterized, accumulating evidence supports the role of TANs in cancer angiogenesis and metastases, thereby contributing to pro-tumor functions. Indeed, infiltrating TANs predicts poor overall survival in many types of cancer. Notably, both their accumulation and specific distribution within tumor sub-regions have prognostic relevance [93]. Anti-tumorigenic TANs can kill tumor cells through oxidative damage-dependent cytotoxicity or Fas-L-induced apoptosis [91,93,94]. Furthermore, TANs secrete key mediators involved in tissue remodeling, including metalloproteinases, such as metalloprotease MMP-9, and elastases, such as neutrophil elastase, which remodel the extracellular matrix and the vasculature tissue [91].

DCs are APCs that play a key role in bridging innate and adaptive immunity. They interact with effector T cells through immunological synapses, thus presenting antigens and triggering their activation. In particular, cDC1, known for their ability to cross-present antigens to activate CD8+ cytotoxic T cells, play a significant role in the recognition of tumor antigens and the initiation of anti-tumor immunity [95]. Because of their crucial role as inducers of effector anti-tumor responses, DCs are key targets of the immunosuppressive TME. Indeed, tumors can subvert DC functions through several mechanisms, including the prevention of direct contact with either tumor cells or effector T cells [96]. Consequently, targeting DC functionality may be an effective strategy to improve anti-tumor therapies [96,97,98].

- T cells

Within the TME, multiple T cell types influence the tumor development. Depending on their differentiation phenotype, CD4+ T cells can regulate several immune responses that are very context-dependent and can either be beneficial or detrimental for immune surveillance. For example, T helper 1 (Th1) CD4+ T cells exhibit a pro-inflammatory function, supporting CD8+ cytotoxic activity. Also, Th17 cells have been described in tumor immune infiltrates, although their function in this setting is still unclear [99]. Notably, T helper subsets exhibit extreme plasticity, allowing them to switch from one subset to another in response to diverse cytokine environments [100].

Regulatory T cells (Tregs), characterized as FoxP3+CD25+CTLA-4+ CD4+ T cells, mostly exert immunosuppressive functions within the TME. These functions may involve suppressing the activity of conventional T cells (including impairing antigen cross-presentation) or secreting pro-angiogenic and immunosuppressive cytokines, such as IL-10. Consequently, their accumulation is often associated with poor cancer prognosis [101,102].

CD8+ T cells differentiate in CTLs, which can attack tumor cells through several mechanisms, such as releasing cytotoxic granules or stimulating death receptor pathways, leading to tumor cell death. In addition to their cytotoxic activity, CTLs can also exhibit anti-angiogenic activity by secreting cytokines that counteract vascular endothelial growth factor (VEGF). Conversely, the accumulation of CTLs in the tumor area is often associated with a favorable prognosis in cancer patients [2,82]. Different CD8+ T-cell subtypes depend on their differentiation state, function, and phenotype. Recently, the importance of a novel subtype, stem-like memory T cells (Tscm), with the potential to generate a diverse range of effector T cells over time has been highlighted [103].

#### 3.1.2. TME Non-Immune Cell Infiltrates

In addition to immune cells, TME cell components also include resident mesenchymal support cells, adipocytes, fibroblasts, neurons, and endothelial cells.

- Cancer-associated fibroblasts (or CAFs)

CAFs are the most prominent stromal components of the TME, with a major contribution to tumor progression through the secretion of extracellular matrix components and remodeling enzymes [104]. CAFs are activated by tumor-derived TGF-β, and they, in turn, produce this growth factor, further contributing to a positive feedback loop. Recent evidence showed that extracellular vesicles from oral cancer cells can also activate fibroblasts into CAFs [105]. Among the various non-tumor cells in the TME, CAFs play multiple roles in cancer, which are not commonly associated with fibroblasts [106]. Moreover, CAFs are key regulators of immune cell recruitment and functions within the TME (see next sections).

- Endothelial cells

Vascular endothelial cells (ECs) are highly specialized cells that line the luminal side of blood vessels and form a selectively permeable exchange barrier between the blood and tissues [107]. The endothelium plays a crucial role in immune cell trafficking during both homeostasis and pathology, including tumor development. Transcriptional profiling has shown that the presence or absence of T cell tumor infiltrates correlated with distinct EC profiles, highlighting active endothelial cell mechanisms regulating immune cell infiltration within the TME [108].

#### 3.1.3. The Extracellular Matrix

The ECM is a non-cellular component of the TME, comprising both fibrous and non-fibrous structures that can vary between cancer types and developmental phases [85]. In particular, the ECM includes the basement membrane (BM), primarily composed of laminins and collagen IV, and the stromal ECM, which consists of glycoproteins such as collagen, elastin, proteoglycans, hyaluronic acid, laminins, and fibronectin. The BM separates endothelial and epithelial cells from the stromal ECM, which provides tissue strength and facilitates remodeling and mechanical signaling [109]. The ECM imbeds and supports neoplastic cells and facilitates adhesion-dependent intercellular communication and cell migration [85,110,111].

The loss of ECM homeostasis, including alterations in topology and biomechanics, is a hallmark of cancer progression [112,113].

### 3.2. Tumor Cell-Driven Hypoxia and Its Effects on Immune Surveillance

Tumor cells can secrete several factors that influence the TME, including cytokines, growth factors, ECM-remodeling enzymes, and metabolites. The varying concentrations of these soluble factors within the microenvironment and the high metabolic rate of cancer cells can alter pH and oxygen levels, thereby influencing a range of cellular players, including immune cells.

Hypoxia is a common hallmark of malignant tumors, driven by several factors, including carcinogenic factors (such as microbiota dysbiosis), high metabolism, excessive cell proliferation within a confined space, vasculature deformation due to pressure, and dysregulated proliferation and alignment of vascular endothelial cells [114].

The hypoxic TME is detrimental to immune surveillance. Indeed, hypoxia stimulates the expression of the hypoxia-inducible factor (HIF)-1α, which in turn can significantly increase PD-L1 expression on TAMs, MDSCs, DCs, and cancer cells within the TME [115,116]. PD-L1 is considered an immunosuppressive ligand because its interaction with the inhibitory receptor PD-1 on T cells leads to decreased activation and cytotoxic functions. Interestingly, PD-L1 expression is driven by the direct binding of HIF-1α to a hypoxia response element in the proximal promoter of the PD-L1 gene, further promoting T cell tolerance [115,117]. In addition to the PD-L1 checkpoint stimulation, TME hypoxia also promotes immune escape through various other mechanisms, as reported elsewhere [118,119,120,121]. These mechanisms include the inhibition of DC stimulatory capacity, downregulation of T cell receptor transduction, induction of Treg cell bias, T cell apoptosis and exhaustion, and the activation of immunosuppressive and tumor tolerance programs. Furthermore, hypoxia can also induce tumor cells to release various immunosuppressive factors, including vascular endothelial growth factor (VEGF) and transforming growth factor-β (TGF-β), chemokines CCL28, CCL20, and prostaglandin E2, which have downstream effects on the function of other immune cells. In addition, hypoxia within the tumor microenvironment significantly impairs T-cell migration and function. Low oxygen levels alter the expression of adhesion molecules and chemokines, disrupting T cell trafficking and reducing their ability to effectively target and kill tumor cells, as reported in a mouse tumor model where T cells were monitored using two-photon microscopy [122]. Hypoxic conditions also lead to the accumulation of inhibitory signals that further dampen T-cell activity.

### 3.3. Role of TME on Ion Channels and Immune Cell Anti-Tumor Functions

Ion channels, serving as vital cellular gateways, influence multiple biological immune cell processes, including intracellular ion homeostasis and integrin stimulation (see Section 2.2), which, in turn, are necessary for immune cell activation, differentiation, and effector functions both in healthy and pathological conditions (e.g., infections, autoimmunity, cancer) [123,124]. Several ion channels are expressed in and regulate the functions of both innate and adaptive immune cells, as extensively reviewed by others [124,125].

Several features of the TME can act on ion channels to inhibit immune surveillance [126]. For example, the TME is characterized by an elevation in the extracellular potassium concentration, which derives from increased local necrosis [127]. Extracellular K^+^ elevation impairs TCR-driven Akt-mTOR phosphorylation, transcriptional programs required for cytokine production, and effector functions [127]. Accordingly, the presence of necrosis has been associated with poor prognosis in several solid malignancies [128]. Furthermore, hypoxia may inhibit the surface expression of the voltage-dependent (Kv) Kv1.3 ion channel [129], thus causing T cell membrane depolarization and altered Ca^2+^ signaling and Ca^2+^ signaling-dependent functions [129,130]. Adenosine, a catabolic product of adenosine triphosphate (ATP), is a crucial immune regulator within the TME [131]. The Ca^2+^-activated K^+^ channel KCa3.1 (also known as IKCa1) mediates immune cell response to adenosine [132,133,134]. In particular, it has been found that KCa3.1 compartmentalizes at the T cell uropod together with TRPM7 (transient receptor potential cation channel subfamily M member 7) and that both channels participate in the regulation of membrane potential and T cell locomotion [134]. Chimote and colleagues have demonstrated that TME adenosine stimulated cAMP production and protein kinase A1 (PKAI) activation, thus inhibiting KCa3.1 channels and suppressing T cell migration in cancer patients [133].

Given the crucial effect of ion channels on integrin-dependent cell-ECM and cell–cell adhesion events [42,45], recent lines of investigation are exploring the potential of altering channel function to improve immune cell capacity to eliminate cancer cells [126,135]. For example, increased activation of the Kv1.3 channel enhances effector T cell anti-tumor activity [136]. Similarly, targeting cancer cells’ ion channels might affect some essential cellular functions and/or increase their susceptibility to chemotherapy and radiation therapy [135,137]. Overall, targeting ion channels can have a combinatorial effect on both tumor cells (e.g., tumor development, drug resistance) and on the operation of immune cells (integrin-dependent functioning). Indeed, treatments targeting ion channels should consider immune cell versus cancer cell specificity and off-target effects. Further understanding of the roles of these channels in cancer immunology is therefore necessary. Likewise, further studies are necessary for characterizing whether and how cancers actively adopt specific mechanisms of subversion of immune cell integrin-ion channel complexes to escape immune cell infiltration and evade immune surveillance.

## 4. Tumor Escape Strategies Targeting Adhesion Cues to Impair Immune Cell Infiltration and Surveillance

The dynamic topography of immune infiltrates into solid tumors strongly depends on adhesive and migratory signals provided by the local TME. Moreover, the timing and extent of immune cell infiltration and trafficking within tumor tissues are essential for eliminating malignant cells and reconstituting tissue homeostasis.

**Figure 2:**
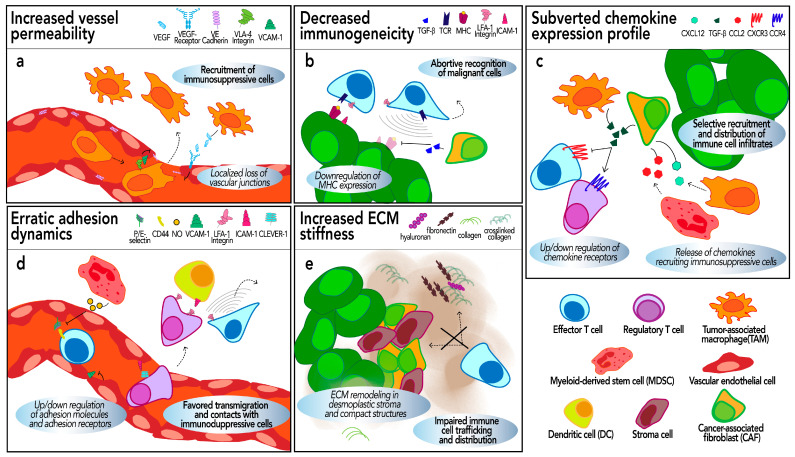
Tumor mechanisms of subversion of immune cell infiltration within the TME. The panels show several mechanisms, discussed in the text, adopted by tumor or tumor-associated cells to evade effector immune cell infiltration. The legends at the top of each panel illustrate the specific structures and molecules involved in each mechanism. Cell movements are represented as dashed arrows. (**a**) TAM-driven secretion of VEGF can reduce the expression of vascular VE-cadherin, causing transient and local leakiness. TAM VLA-4 interaction with VCAM-1 on vascular endothelial cells favors further TAM diapedesis. (**b**) Loss of MHC-I expression due to CAF-secreted TGF-β makes tumors less visible to the immune system, affecting effector T cell infiltration and tumor control. (**c**) TGF-β produced by TAMs or CAFs enhances the expression of CCR4 in Tregs, whereas it decreases the CXCR3 expression on CD8 T cells, thus promoting the infiltration of the first ones over the latter. CAF and/or MDSC secrete CXCL12 and CCL2 to recruit TAMs and monocytes, respectively, in the TME. (**d**) MDSCs production of NO downregulates the expression of CD44 and P/E selectin, impairing effector T cell extravasation and tumor infiltration. Higher expression of LFA-1 integrin on the surface of Tregs favors their interaction with APCs, leading to decreased activation of effector CD8+ T cells. Increased expression of CLEVER-1 (see Section: Mechanisms of Subversion of Adhesion Molecules) on the surface of vascular endothelial cells promotes Tregs diapedesis through ICAM-1 in the TME. (**e**) CAF deposition of collagen and other ECM components increases the tumor stiffness, hindering effector T cell migration and distribution within the TME. CAF organization with stroma cells in desmoplastic reactions surrounding tumor cells creates a physical obstacle limiting effector immune cell trafficking.

Tumors can adopt several strategies to prevent immune cell infiltration, thus impeding anti-tumor immune functions (summarized in Figure 2). Indeed, the inefficient tumor infiltration by immune cells, particularly cytotoxic T cells, represents one of the most important limiting factors for the efficacy of immunotherapies, such as vaccines and adoptive cell transfers [138,139]. Moreover, cancer patients with tertiary lymphoid structures (TLSs) and lymphoid aggregates tend to have more favorable outcomes and responses to immunotherapies [79,80]. Thus, novel immune strategies for cancer treatment aim to overcome the barriers of immune cell access to tumors and empower the effectiveness of immune infiltrates [140,141,142,143].

The TME plays a pivotal role in regulating the immune contexture, and mediating strategies that exclude immune infiltrates from the vicinity of cancer cells [144]. Here, we examine the mechanisms by which the tumors evade immune cell trafficking and infiltration within the TME, particularly those that rely on immune cell adhesion (Figure 2). To highlight the importance of these mechanisms, we also provide a few examples of their use for the improvement of immunotherapies.

### 4.1. Dysfunctional Tumor Vasculature Alters Immune Cell Accessibility to the TME

Angiogenesis is a key hallmark of tumorigenesis, implying pre-existing blood vessels forming new ones in order to provide more nutrients for the cancer cells and facilitate their dissemination throughout the body. Notably, tumor angiogenesis encompasses the development of excessive and dysfunctional vessels [145,146]. Indeed, tumor vasculature is often morphologically aberrant, characterized by dilated and fragile vessels, increased leakiness, and reduced pericyte coverage [147,148]. These features not only significantly impede the delivery and distribution of anti-cancer drugs [149], but are also strongly detrimental to immune cell functions [147,148]. For example, compromised hemodynamic stability and increased vessel permeability might impair the delivery of oxygen and nutrients to the TME, further exacerbating hypoxia and hypoxia-driven immune cell dysfunctions. Moreover, the alteration in the blood vessel diameter can impair the ability of immune cells to tether and/or transmigrate across the vasculature, thus reducing their infiltration into the TME.

Numerous regulators are engaged in tumor angiogenesis. Among them, the VEGF/VEGFR signaling pathway is dominant in the vasculature system, and it is indeed the main focus of anti-angiogenic tumor therapies [146,150]. Among the components of the VEGF family, VEGF-A and its receptors VEGFR-1 and VEGFR-2 play major roles in both physiological as well as tumor angiogenesis [151]. HIF-1α is one of the main activators of VEGF, as well as of other pro-angiogenic factors including TGF-β, fibroblast growth factor (FGF), platelet-derived growth factor (PDGF), nitric oxide synthase (NOS), and interleukin-8 (IL-8) [152,153,154]. HIF-1α overexpression in tumors is also coupled with the downregulation of phosphatase and tensin homolog (PTEN), which was associated with vasculature hyperplasia [155].

The HIF/VEGF signaling pathway can be activated simultaneously in both tumor cells and cancer-associated immune cells, providing advantageous conditions for tumor cells to intravasate and invade distant sites and for cancer-associated immune cells to circulate more effectively in situ [156,157]. Moreover, within the same TME, the expression of the HIF/VEGF signaling pathway in cancer cells versus immune cells may differ, potentially favoring one population over the other. For example, the HIF-2α protein expression in TAMs concerns the majority of uterine cervical cancer specimens, whereas its expression in tumor cells was found in less than 10% of the same specimens [158]. TAMs expressing HIF-2α have also been identified in various types of human tumors, including lung, urothelial, bladder, and breast cancers [159,160,161,162]. Therefore, TAMs act as active regulators of the tumor microvessel permeability, facilitating their own access and circulation within the tumor niche. Of note, the frequency of HIF-2α+/VEGF+ TAMs in breast carcinomas has been associated with reduced overall survival rates [161]. Furthermore, a higher percentage of such TAMs in tumor patients correlates with a more unfavorable prognosis and a lower probability of survival following chemotherapy [158,160,162].

Despite numerous reports about the role of TAMs in promoting tumor vasculature permeability, the underlying mechanisms remain poorly understood. TAMs appear to directly target the ECs of the blood vessels. On the one hand, the direct secretion of VEGF (or exosomes containing VEGF and other pro-angiogenic factors [163]) by TAMs reduces the expression of the vascular junction proteins such as Zonula Occludens-1 (ZO-1) and VE-cadherin in ECs, leading to transient and localized loss of vascular junctions [156] (Figure 2a). On the other hand, the direct interaction between VLA-4 on M2 macrophages and VCAM-1 on ECs ultimately regulates the downstream RAC1/ROS/p-PYK2/p–VE-cadherin pathway, resulting in increased vessel leakiness [164] (Figure 2a).

Overall, emerging evidence highlights the major role played by TME-associated abnormal and dysfunctional blood vasculature in thwarting immune cell infiltration dynamics. As mentioned above, these conditions prevent cancer infiltration by anti-tumor immune cells while favoring the infiltration of TAMs and other immunosuppressive populations [165,166]. Hence, targeting vasculature normalization could enhance both an anti-tumor effective immune cell contexture and the therapeutic efficacy of cancer immunotherapy [150,154,165]. Vascular normalization strategies include targeting the VEGF/VEGFR pathway. Anti-VEGF therapy has been shown to improve chimeric antigen receptor (CAR)-T cell homing and distribution into the TME and delay tumor progression in a murine glioblastoma model [167]. Several VEGF/VEGFR-targeting therapies are in development. Apatinib, a selective inhibitor of VEGFR-2, is currently undergoing phase II clinical trials for several solid tumors, including hepatocellular carcinoma (NCT04191889) [168], gestational trophoblastic disease (NCT04047017) [169], esophageal squamous cell carcinoma (NCT03603756) [170], and liver cancer (NCT03092895) [171]. Some of these treatments, summarized in Table 1, are in combination with chemotherapy and/or PD-L1 immunotherapy. Among those therapies targeting VEGFR-1/2/3, sulfatinib, also known as surufatinib, has been evaluated in phase III (NCT02588170), demonstrating clinical efficacy in patients with advanced neuroendocrine tumors [172] (Table 1). Likewise, fruquintinib treatment has shown increased overall survival in patients with metastatic colorectal cancer in a phase III clinical trial (NCT02314819) [173] (Table 1). Furthermore, the combination of PD-1 and VEGFR-2 blockade increased PD-1 expression in tumor-infiltrating CD4+ T cells, and those cells were able to promote vasculature normalization in hepatocellular carcinoma models [174]. Because immune checkpoint blockade combined with sorafenib (a pan-VEGFR inhibitor) has led to improved immune cell infiltration and productive anti-tumor immunity in hepatocellular carcinoma (HCC) patients [175], several combination regimens have been approved by the Food and Drug Administration (FDA) for clinical trials, and the concept of combining vascular normalization therapy with immunotherapy is currently in clinical practice [150,176]. Preclinical results in small-cell lung cancer, colorectal cancer, and breast cancer suggest that vasculature normalization by inhibiting the VEGF/VEGFR pathway reprograms the immunosuppressive tumor microenvironment and enhances immunotherapy [177,178,179,180]. Multiple agents have been approved in combination with PD-L1 therapy and have been recently reviewed by Patel and colleagues [181]. Interestingly, the lenvatinib/pembrolizumab combination is showing consistent and durable benefit and overall good safety in an ongoing phase III clinical study in patients with advanced renal cell carcinoma (NCT02811861) [182] (Table 1). Other anti-panVEGFRs have shown successful efficacy in the treatment of solid tumors, in combination or not with PD-L1 therapy and/or chemotherapy. For example, bevacizumab has shown promising results in a phase III clinical trial in women carrying platinum-resistant/refractory ovarian cancer (NCT05281471) [183] or advanced ovarian cancer (NCT03038100) [184]. Finally, Cui and colleagues have developed a new bispecific antibody, termed HB0025, targeting both VEGFR1 and PD-L1 [185]. This dual-target “biotherapy” has demonstrated higher efficiency in inhibiting cancer growth than the monotherapies targeting the two molecules in preclinical setups [185]. Overall, given the increased amount of treatments approved for routine clinical use, vasculature normalization has gradually gained more attention in the progress of anti-tumor immunotherapies.

### 4.2. Reduced Immunogenicity Makes Cancer Less Visible to the Immune System

Major histocompatibility complexes, including MHC class I (MHC-I) and class II (MHC-II), have crucial roles in cell-intrinsic and pathogen-derived antigen presentation, respectively. MHC-I is distributed on the surface of almost all nucleated cells and is necessary for the presentation of endogenous antigens to effector T cells. MHC-I loss is a frequent mechanism of immune escape. An MHC-low phenotype has been found in many human cancers, such as breast and melanoma [191]. This loss of MHC-I expression renders tumors less visible to the immune system, preventing T cell recognition of tumor antigens and, consequently, reducing T cell efficacy in controlling cancer.

Expression of MHC is necessary for the recruitment of immune cells for tissue patrolling (Figure 2b). Conversely, cancers with low MHC-I expression (e.g., breast cancer) typically contain fewer infiltrating T cells compared to their MHC-I-high counterparts [191,192,193,194]. Likewise, a study carried out on a cohort of stage III melanoma samples [195] showed that high MHC-I expression was associated with a greater number of T cell infiltrates and longer overall survival [195,196,197]. Tumors adopt several mechanisms to escape MHC-driven immune cell infiltration. Hypoxia and HIF expression within the TME can potentiate immune escape by downregulating the expression of the antigen-presenting MHC-I complexes on malignant cells [198,199]. CAF-derived TGFβ could also reduce MHC-I expression in ovarian cancer cells in vitro [200] (Figure 2b).

MHC re-expression represents a key strategy for future immunotherapies. Conversely, monitoring its expression during cancer progression can inform the outcome of T-cell-mediated cancer therapy [201]. Some toll-like receptor agonists have been demonstrated to induce antigen-presenting properties by non-APC-derived tumor cells. For example, the Bacillus Calmette-Guerin (BCG), an agonist of TLR2 and TLR4, is approved by the US FDA for immunotherapy of bladder cancer [202]. This has been shown to increase the expression of MHC-II, CD80, and ICAM-1 on the surface of epithelial cancer cells [202].

### 4.3. Altered TME Cytokine/Chemokine Secretion Profile Misinstructs Immune Cell Recruitment and Dynamics Within Malignant Niches

Chemokines are crucial for the recruitment and trafficking of both stimulating and suppressive immune cell types in the TME. The pattern of chemokine-receptor signaling in diverse tumors correlates with the density and type of immune cell infiltrates, and dysregulation of this signaling has been correlated with mechanisms of tumor escape and progression [8,23,203].

Despite the large variety of chemokines and chemokine receptors regulating immune cell behavior and dynamics in cancer, we outline below the major characterized chemokine axes associated with tumor mechanisms of immune escape. Additionally, we provide some key examples of drug treatments and cellular immunotherapies, highlighting the significance of chemokine and chemokine receptors for cancer treatments.

#### 4.3.1. Mechanisms of Tumor Harnessing of the Immune Cell CXCR4-CXCL12 Axis

The CXCR4-CXCL12 axis was first identified as a regulator of lymphocyte trafficking and retention in the bone marrow. However, CXCL12 is expressed at high levels in bladder cancer, gastric cancer, prostate cancer, and many other human tumors [204,205,206,207,208].

Increased secretion of CXCL12 has been associated with tumor chemorepellent activity towards antigen-specific T cells and with escape from immune control in melanoma and in pancreatic ductal adenocarcinoma (PDA) murine models [209,210]. Likewise, high CXCL12 correlated with poor overall and disease-free survival in stage II PDA patients [211]. Overexpression of CXCR4 has been identified as a poor prognostic marker in various tumors, such as colorectal, gastrointestinal, breast, and prostate cancer [212,213,214,215] (Table 2).

CAFs were found to be the principal source of CXCL12 in a PDA murine model [210]. Interestingly, a study by Feig et al. demonstrated that CAFs coat cancer cells with CXCL12, thus favoring reciprocal interaction between the two cell types. Therefore, CXCL12-coated cancer cells surrounded by CXCR4-expressing CAFs inhibited both CTL access to the TME and their interaction with malignant cells. On the other hand, CXCR4 blockade promoted T cell infiltration into the tumor, increased T cell anti-tumor activity, and improved immunotherapy treatments [187,210,216].

Furthermore, two distinct studies carried out in breast and colorectal cancer samples showed that CAF secretion of CXCL12 recruited monocytes to the tumor area and further induced their differentiation into M2-like macrophages (Figure 2c). These TAMs in turn produced the anti-inflammatory cytokine IL-10 and suppressed NK cell functioning, thus promoting an immunosuppressive environment [217,218].

Overall, the activation of the CXCR4-CXCL12 axis is a key tumor immune escape mechanism, as it prevents the infiltration of effector T cells while promoting the infiltration of immunosuppressive myeloid populations. Consequently, this axis has emerged as a promising target for cancer therapeutics [23,219]. AMD3100 (commercially known as plerixafor) is an FDA-approved specific antagonist targeting CXCR4. However, because of drug resistance, some pre-clinical studies have proposed a combination of AMD3100 with Bortezomib, the latter being more efficient on AMD3100-treated poorly adherent cancer cells [186,220] (Table 2). A phase II clinical study in multiple myeloma patients demonstrated that the combination of AMD3100 with bortezomib is more effective at killing tumors than either treatment alone (NCT00903968) [186] (Table 1). AMD3100 treatment has been mostly used for blood malignancies. However, preclinical studies in prostate, colon, breast, and small cell lung cancer have shown that AMD3100 has an inhibitory function on both cancer growth and metastasis [221,222,223,224]. Furthermore, AMD3100 treatment in an in vivo model of estrogen receptor-positive breast cancer reversed tamoxifen resistance, indicating that the combination of anti-CXCR4 with endocrine therapy could be a more efficient approach for the treatment of this type of tumor [225]. Interestingly, blocking CXCR4 by AMD3100 increased T-cell infiltration in mouse models of breast cancer with high efficacy when combined with anti-PD-1 and anti-CTLA-4 treatments [224]. In addition to AMD3100, other anti-CXCR4 antibodies have shown efficacy in solid tumors. For example, 12G5 has demonstrated anti-tumor and anti-metastatic activity in both endometrial cancer and osteosarcoma xenograft mouse models [226,227]. Similarly, among the anti-CXCR4 peptide antagonists, motixafortide, balixafortide, and LY2510924 have been evaluated in clinical trials. Treatment of pancreatic cancers with motixafortide, combined or not with chemotherapy, has shown clinical success in a phase II clinical trial for pancreatic cancer (NCT02826486) [187]. Balixafortide treatment in combination with chemotherapy has been tested in a phase I clinical trial in patients with heavily pretreated, relapsed metastatic breast cancer (NCT01837095) [188]. Finally, despite successful pre-clinical studies, the addition of the anti-CXCR4 peptide LY2510924 to cancer therapy did not improve the efficacy of the treatments in phase II studies in lung cancer and renal cell carcinoma, respectively NCT01439568 [189] and NCT01391130 [190]. Hence, further studies are evaluating the anti-cancer activity of both LY2510924 and new compounds targeting the CXCR-CXCL12 axis. For example, the CXCR4 antagonist IS4 has been shown to reduce tumor metastasis in prostate and melanoma cell lines [228].

Overall, this significant preclinical and clinical evidence raises interest in CXCR4 treatments to inhibit cancer progression, in combination or not with chemotherapy and/or checkpoint inhibitor therapies.

**Table 2:** Summary of the main cell adhesion molecules, chemokines, and their receptors mentioned in the review and represented in Figure 1: prognostic outcome, functions, and immunotherapy implications.

Chemokines—Receptors	Prognosis: Cancer Type	Mechanisms of Action	Immunotherapy Treatments
CXCL12- CXCR4	**Poor prognosis**: PDA [211], breast cancer [215], lung adenocarcinoma, gastrointestinal cancer [212], prostate cancer [213]	CAFs coat cancer cells with CXCL12, excluding CTL from the tumor [210]; CAF-derived CXCL12 recruits monocytes to the tumor area [217]	Inhibition of CXCR4-CXCL12 mobilizes CD8+ T cells in the tumor and synergizes with anti-PD-L1 immunotherapy [210,216]; CXCR4 antagonist AMD3100 is used to improve efficacy of bortezomib treatment [186] and anti-PD-1 and anti-CTLA-4 therapy in breast cancer mouse models [224].
CXCL9/10/11-CXCR3	**Good prognosis**: Gastric cancer [229] **Poor prognosis**: Breast cancer [230], colon cancer [231]	Effector immune cells NKs and CTLs accumulate via the CXCR3-CXCL9/10/11 axis [232]; CAF-driven down-regulation of CXCR3 in pancreatic tumor cultures inhibits T cell tumor infiltration towards CXCL10 [233]; M2-derived TGF-β inhibits CTL surface expression of CXCR3 and trafficking in a murine model of colorectal cancer [234]	CXCR3 chemokine axis is required for the efficacy of anti-PD-1 therapy [16,235]
CCL17/CCL22-CCR4	**Good prognosis**: Lung cancer [236], melanoma [237], head and neck squamous carcinoma [238] **Poor prognosis**: Renal cancer [239], testicular cancer [240]	TAM, or cancer cell-derived CCL22, associates with higher CCR4+ Treg infiltration [241,242]	CCR4 antagonist mogamulizumab decreases Treg immunosuppressive activity [243]; combination of mogamulizumab with anti-PD-1 antibody depletes Treg and increases CD8+ tumor infiltration [244]
**Cell adhesion molecules**	**Prognosis: Cancer type**	**Mechanisms of action**	**Immunotherapy treatments**
VCAM-1/VLA-4	**Poor prognosis:** Renal [245], gastric [246], and ovarian cancers [247]	VCAM-1 overexpression increases monocyte infiltration [248] and promotes interaction between cancer cells and TAMs [218]	Blockade of VCAM-1-VLA-4 interaction reduces pulmonary osteosarcoma incidence in preclinical mouse models [249]; CAR T cell against VEGFR2 increases VCAM-1 expression on vascular ECs [250]
ICAM-1/LFA-1	**Poor prognosis:** TN-breast [251]; colorectal [252]; gastric cancer [253]; thyroid [254]; melanoma [255]	ICAM-1 downregulation protects from NK- and CAR-T-mediated cytotoxicity in breast cancer and carcinoma cells [256,257]; CTL lysis in melanoma metastasis [255]	Engineered CAR-NK cells overcome ICAM-1 reduction in breast cancer [256]; ICAM-1 CAR T cells mediate profound tumor killing of ATC tumors [258]

#### 4.3.2. Mechanisms of Tumor Sabotage of the CXCR3-CXCL9/10/11 Axis

The CXCR3-CXCL9/10/11 axis is one of the most potent signaling axes in anti-tumor immunity. It was shown to be necessary for the recruitment of cytotoxic T cells, NK cells, and M1 macrophages in several murine models, including lymphoma, renal cell carcinoma, melanoma, and breast cancer [23,232,259,260,261]. The chemokine receptor CXCR3 is predominantly expressed on the surface of macrophages, T cells, NK cells, dendritic cells, and cancer cells. The main sources of its ligands, CXCL9, CXCL10, and CXCL11, are macrophages, endothelial cells, fibroblasts, and cancer cells. CXCL9, CXCL10, and CXCL11 play distinct roles in tumorigenesis [262]. Experimental evidence across different tumor models indicates that the deficiency of these three CXCR3 ligands significantly impairs effector immune cell recruitment and cell-mediated anti-tumor functions. The accumulation and retention of both NKs and cytotoxic T cells in tumors are dependent on the CXCR3-CXCL9/10/11 axis and are correlated with prolonged survival and enhanced efficacy of immunotherapy in murine models of myeloma, lymphoma, and melanoma [9,260,263]. High expression of CXCR3 has been correlated with a better prognosis of gastric cancer [229]. However, high CXCR3 expression in melanoma, colon, and breast cancers has been associated with more malignant and aggressive tumors [230,231,261].

The CXCR3-CXCL9/10/11 axis plays a dual regulatory role in cancer. On the one hand, it is associated with the development and progression of many tumors, including increased invasiveness. On the other hand, it can inhibit tumor growth by promoting the infiltration of effector immune cells within the tumor [261]. Despite the well-documented role of this pathway in immune cell infiltration, there is limited experimental evidence suggesting that the subversion of either CXCR3 or its ligands favors tumor immune escape. A study by L. Gorchs and colleagues showed that CAFs down-regulate the expression of CXCR3 and CCR5 while upregulating CXCR4 expression in both 2D and 3D pancreatic tumor cultures, thereby inhibiting T cell tumor infiltration towards CXCL10 [233]. Furthermore, immunosuppressive macrophages from patients with ErbB-mutated gallbladder cancer up-regulated the expression of CXCL10, which in turn favored the interaction with Treg cells, thus disabling anti-tumor immune activity [264]. Finally, the immunosuppressive TME may prevent immune cell infiltration through the release of cytokines targeting the CXCR3-CXCL9/10/11 axis. For example, TGF-β produced by CAFs or M2 macrophages in the TME was found to inhibit the expression of CXCR3 on the surface of cytotoxic T cells in a murine model of colorectal cancer (Figure 2c). Conversely, TGF-β receptor I-deficient CD8+ T cells exhibited increased CXCR3 expression and enhanced trafficking into tumors [234]. Similarly, BLT1(−/−) and CXCR3(−/−) mice showed a significant reduction in tumor-infiltrating CD8+ T cells as compared to their wild-type counterpart, despite similar frequencies of these cells in the periphery [260].

In addition to the quite complex biological behavior of CXCR3 and its ligands in tumor immunity, diverse CXCR3 splice variants exist, and they can be differently expressed in different cell types. As such, several immunotherapies have shown unsuccessful results. For example, the CXCR3 neutralization in mc38 tumor-bearing mice diminished the efficacy of the PD-1/PD-L1 combinatorial immunotherapy by fostering CD8+ T cell exclusion from the TME [235]. In contrast, in another successful application of anti-PD-1 immunotherapy, Chow et al. have shown that the CXCR3 axis is a biomarker for the sensitivity to PD-1 blockade and that augmenting its TME activity could improve clinical outcomes [16] (Table 2).

#### 4.3.3. Mechanisms of Tumor Harnessing of the CCR4-CCL17/CCL22 Axis

The CCR4 chemokine receptor is mainly expressed by Th2, Th17, and Treg cells. Its ligands, CCL17 and CCL22, are mainly released by tumor cells and TAMs. CCR4 plays an essential role in other adverse biological behaviors, and its expression or the one of its ligands has been associated with improved prognosis in lung cancer, head and neck squamous cell carcinoma, and melanoma patients [236,237,238], and poor prognosis in patients with renal cancer and testicular cancer [239,240].

Interestingly, since the expression of CCR4 is higher in Tregs than other CD4+ T cells, probably because of a FOXP3 responsive element on the CCR4 promoter [265], Tregs infiltration is favored in some tumors, e.g., in breast cancer [241]. The selective recruitment of this cell population, by either increased CCR4 expression or ligand production, may hence represent a mechanism by which tumors may favor an immunosuppressive TME [240,241,265,266]. In fact, CCL22 produced by TAMs and ovarian tumor cells was associated with higher Treg infiltration in breast cancer, gastric cancer, and squamous cell carcinoma [241,242]. In breast cancer patients, TGF-β produced by CAFs or M2 macrophages promoted the differentiation of CD4+ T cells into Tregs (FOXP3+ CD4+). Furthermore, TGF-β-induced FOXP3 expression enhanced CCR4 expression in Tregs, thereby promoting their infiltration into tumors and fostering immune privilege [265] (Figure 2c). Given that Tregs are functionally immunosuppressive, their presence within tumors is often associated with poor prognosis [242,267,268]. Apart from Tregs, CCR4 blockade has been shown to decrease the accumulation of immature myeloid cells in the TME, augmenting the survival of syngeneic pancreatic cancer-bearing mice [269].

Both mogamulizumab, a humanized anti-CCR4 monoclonal antibody, and CCR4-CAR T therapy have shown great efficacy for the treatment of blood malignancies [270,271]. In a clinical trial, mogamulizumab treatment of solid tumors reduced the amount of CD4 T cells in the peripheral blood, including Tregs, with minor effects on effector CD8 T cells. Additionally, almost half of the patients have shown enhanced immune responses and increased long-term survival [243] (Table 2). Finally, a phase I clinical report of mogamulizumab in combination with anti-PD-1 treatment in patients with advanced or metastatic solid tumors has found a clear depletion of effector Tregs and tumor-infiltrating CD8+ expansion. This has correlated with enhanced anti-tumor immune responses and a good safety cancer patient profile [244] (Table 2).

#### 4.3.4. Tumor Manipulation of Other Chemokine Signaling Pathways

Cytokine release in the TME can also result from genetic alterations. For instance, CCL9 secretion by Myc-induced lung adenocarcinoma promotes the recruitment of macrophages to the TME [272], whereas oncogenic KRAS upregulates the production of GM-CSF in pancreatic ductal cells, which in turn favors the infiltration of immunosuppressive myeloid cells [273].

CAFs can acquire anti-inflammatory expression signatures characterized by the upregulation of immunomodulatory molecules, such as TGF-β or PD-L1/L2, as well as chemokines, such as CXCL12, CCL2, CCL3, CCL4, and CCL5. CCL2 upregulation in CAFs in a murine liver tumor model recruited MDSC in the tumor area [274]. Conversely, CCL2 upregulation favored the recruitment of Tregs in lung cancer [275]. Conversely, the transduction by lentiviral vector of the CCL2 chemokine receptor CCR2b into CAR-T cells has led to increased T cell infiltration in malignant pleural mesotheliomas and enhanced anti-tumor activity [276]. Also, CAR-T cells modified with CCR2b have shown increased intratumoral infiltration in subcutaneous human neuroblastoma xenografts in SCID mice, accompanied by reduced tumor growth [277].

The CXCL8-CXCR1/2 axis is upregulated in several cancers, where the secretion of its ligand by tumor-associated epithelial cells, macrophages, or endothelial cells is responsible for the recruitment of N2 TANs and TAMs, as well as cancer stem cells, thereby favoring tumor progression [278,279].

Finally, CAFs were found to inhibit the recruitment of effector CD8+ T cells via the secretion of IL-6 [280].

### 4.4. Inhibition of Adhesion Molecule Expression Impairs Leukocyte Migration Modes and Cell-to-Cell and Cell-to-ECM Interactions

Adhesion molecules and their ligands are pivotal regulators of immune cell infiltrates and can be a potential target of precision cancer immunotherapy [281]. Tumor-associated cells can often modulate the expression of these molecules to completely impede access of immune cells in the tumor site and/or preferentially recruit some immune cells over others (i.e., modulate the ratio CTLs/Tregs). Furthermore, several reports have shown that tumors can adopt various strategies to subvert adhesion-driven IS formation and function to evade anti-tumor effector functions [282,283,284]. For example, tumors can selectively up or down regulate the expression of adhesion molecules on the surface of specific cells to promote the formation of immune-modulatory ISs, such as those between Tregs and CAFs, instead of anti-tumor ISs [284]. Alternatively, they may favor unstable synapses by lowering the antigenic sensing within the microenvironment [283] (see Section 4.2). Nevertheless, given their high and frequent expression in diverse malignant tumors, adhesion molecules have received considerable attention as a prognostic marker [251,285,286].

Herein, we review the mechanisms adopted by tumor cells and tumor-associated cells to dampen adhesive interactions that facilitate effector immune cell interactions with the vasculature and cells within the TME. Furthermore, we provide some examples of cancer treatments employing these adhesion cues to improve overall survival and/or to inhibit immune escape.

#### Mechanisms of Subversion of Adhesion Molecules

- Escape mechanisms targeting VCAM-1 and ICAM-1

Several adhesion molecules expressed on the surface of the vasculature endothelium were reported to be crucial in the different steps of the adhesion cascade necessary for immune effector cells, such as cytotoxic CD8+ T cells, to infiltrate the TME (Figure 1). Among these, the controlled expression of the E/P-selectin and ICAM-1 was described to mediate CTL extravasation [287]. Furthermore, VCAM-1 and CXCL9/10 expressed on the surface of the vasculature endothelium enabled VLA-4+/CXCR3+ CD8+ T cells to access tumors [288]. Several studies have shown that tumor-associated endothelial cells often downregulate the expression of these adhesion molecules, therefore dampening the recruitment and infiltration of effector immune cells [108,287,289]. Hypoxia within the TME seems to play a pivotal role in impairing the expression of both VCAM-1 and of diverse adhesion molecules such as E-selectin, P-selectin, and ICAM-1 on the surface of tumor cells or tumor-associated cells [119,290,291]. The binding of α4β1 integrin-expressing leukocytes to VCAM-1 is indeed necessary for their firm adhesion and further transmigration across the endothelium. As mentioned above, reduced T-cell infiltration significantly correlated with poor survival rates [292,293]. Indeed, VCAM-1 density has been correlated with infiltration of adoptively transferred T cells and with response to checkpoint blockade (PD-L1) in a murine colon carcinoma model [38]. Upregulation of VCAM-1 on endothelial cells of the tumor vasculature has been achieved upon the development of CAR T cells against VEGFR2 [294,295].

In contrast with these studies, another line of evidence indicated that VCAM-1 is overexpressed in several types of tumors, including renal, gastric, and ovarian cancers, where it is likely correlated with angiogenesis, cancer invasion, and metastasis [36,245,246,247,296]. The release of soluble VCAM-1 was reported to enhance the growth and invasion of cancer cells through the activation of the α4β1 integrin [218,297]. Overexpression of VCAM-1 led to an increased monocyte infiltration [248] and promoted interaction between cancer cells and TAMs [218]. One proposed mechanism involves the production of IL-6 by cancer-associated fibroblasts (CAFs), which subsequently upregulates the expression of VCAM-1 on the surface of tumor cells. This recruitment of TAMs promotes an immunosuppressive TME [218].

ICAM-1 is involved in leukocyte cell adhesion, motility, and immune activation at the IS (see Section 2.2.2). Low ICAM-1 has been correlated with poor prognosis in triple-negative breast cancer, where low-ICAM1 was found to promote macrophage M2 polarization along with T-cell exhaustion [251]. Interestingly, ICAM-1 downregulation on breast cancer cells is a key escape mechanism from trastuzumab-triggered NK-mediated cytotoxicity, hampering the ability of these cells to establish productive ISs [256]. Likewise, in solid tumors, low ICAM-1 expression limits EGFR CAR T cells’ ability to form productive synapses, a limitation that can be rescued with IFN-γ, leading to ICAM-1 upregulation [257]. However, engineered CAR-NK cells have been described to overcome cancer cell resistance caused by ICAM-1 reduction because the CAR-mediated killing by NK-92/5.28.z cells used in this study is ICAM-1 independent [256]. On the other hand, ICAM-1 up-regulation has been often correlated with cancer progression, invasion, and immune escape, as in the case of gastric, colorectal, thyroid, and melanoma cancers [252,253,254,255]. Interestingly, metastatic melanoma cells escape CTL-mediated killing by shifting the ICAM-1 expression, which in turn has been associated with activation of the PI3K/AKT-driven signaling contributing to resistance to apoptosis signals [255,298]. Thus, a study from Min et al. has shown that targeting ICAM-1 by CAR-T therapy exhibited big therapeutic efficacy and survival benefits [258]. Furthermore, targeting ICAM-1 along with an anti-PD1 antibody has resulted in rapid tumor clearance and prolonged survival in a mouse model of advanced thyroid cancer [299,300].

- Escape mechanisms targeting CLEVER-1, ETbR, and EpCAM

Endothelial cells from the hepatocellular carcinoma vasculature, upon stimulation by the hepatocyte growth factor, can selectively promote the recruitment of immune suppressive Treg populations. Evidence suggests that this mechanism is mediated by the increased endothelial expression of the common lymphatic endothelial and vascular endothelial receptor 1 (CLEVER-1), which is a scavenger receptor with adhesive activities. CLEVER-1 promotes CD4+ FoxP3+ Tregs transmigration through ICAM-1 and vascular-associated protein-1 (VAP-1) and further infiltration within the tumor niche [301] (Figure 2d). Interestingly, CLEVER-1 is also expressed on the surface of immunosuppressive macrophages, and the density of TAMs positive for this adhesion molecule correlates with reduced overall survival in urothelial bladder, breast, and lung cancer [302,303,304,305]. Indeed, CLEVER-1 was described to prevent the recruitment of TAMs in the TME via a mechanism involving the binding to its ligand stabilin-1-interacting chitinase-like protein (SI-CLP). SI-CLP-mediated inhibition of monocyte migration in the TME was associated with the alteration of CCL2-CCR2-induced cytoskeletal rearrangements, necessary for cell locomotion [305]. Finally, inhibition of CLEVER-1 led to the TAM’s acquisition of pro-inflammatory features and enhanced antigen-presentation activity and responses of cytotoxic CD8+ T cells [304].

Finally, tumor-induced overexpression of two additional adhesion receptors was associated with reduced T-cell infiltration within the tumor bed. R.J. Buckanovich and colleagues demonstrated that the signaling through the endothelin B receptor (ET_B_R) blocked the T cell adhesion to the endothelium, in part via the modulation of ICAM-1. Interestingly, ET_B_R upregulation was also observed in several cancers and correlated with poor prognostic outcomes [108]. Epithelial cell adhesion molecule (EpCAM) is an epithelium-specific intercellular adhesion molecule, highly expressed in a large variety of tumors [285]. A recent study from M. Kanahori and colleagues showed that up-regulation of this adhesion molecule in lung metastases correlated with decreased infiltration of CD8+ T cells [71].

- Escape mechanisms targeting integrins and selectins

Little is known about the escape strategies adopted by solid tumors to affect integrin and selectin-dependent functions. We summarize here some studies correlating the altered expression of selectins and integrins with increased/decreased leukocyte infiltration and tumor outcome. However, further investigations are necessary to address the mechanisms involved in these processes.

Integrins and selectins are both necessary for the adhesion cascade, allowing immune cells to infiltrate underlying tissues. As such, the expression of these adhesion molecules correlated with the recruitment and/or retention of leukocytes in the tumor tissue. αLβ2, α4β1, and αEβ7 integrins contribute to CD8+ T-cell recruitment, infiltration, and killing functions [306,307]. For example, the expression of the αEβ7 integrin was associated with the accumulation of tissue-resident memory T (T_RM_) cells, a specific subset of activated, tumor-reactive CD8+ T cells [308]. Furthermore, high levels of αEβ7 CD8+ T cell infiltration were observed in lung tumors and correlated with improved overall survival in patients with head and neck cancers [309,310,311].

In contrast, expression of the α5 integrin was associated with poor prognosis and correlated with CD4+ T-cells and M2-macrophage infiltrates in gastric cancer [312]. While TAMs might be involved in the regulation of the α5 integrin, MDSCs-driven production of NO could downregulate the expression of the CD44 and PSGL-1 (CD162), therefore impairing T cell extravasation and tumor infiltration [313] (Figure 2d).

Finally, as previously mentioned, integrins not only play a critical role in orchestrating leukocyte migratory behaviors but also serve as immune cell-binding targets for APC-driven activation. Finally, they are also necessary for mediating and enhancing TCR-dependent target cell killing. Thus, functional studies provide evidence that a higher expression of αLβ2 integrin favored the formation of immunological synapses between Tregs and APCs, leading to decreased activation of conventional T cells, such as effector CD8+ T lymphocytes. Consequently, anti-tumor immunity was compromised [314] (Figure 2d).

### 4.5. Increased Tumor Stiffness Mediates Immune Exclusion from the TME

Tumors can adopt a wide variety of tissue architectures resulting from the unique interaction between tumor cells and the surrounding non-cellular components of the ECM. In this way, they can modulate cancer progression and patient survival. TME hypoxia can drive the HIF-dependent transcription of several genes encoding proteins, such as the metalloproteinases MMP-2 and MMP-9, that modulate ECM remodeling [154]. Hypoxia-induced HIF can also promote the synthesis of ECM proteins, such as fibronectin, and the expression of lysyl-oxidases, crosslinkers of ECM components. Overall, hypoxic signaling engages multiple mechanisms promoting ECM stiffness and remodeling [315,316]. Myeloid cells can also modulate cancer stiffness. TAMs stimulated CAFs to produce collagen, thereby directing the deposition, cross-linking, and linearization of collagen fibers at various stages of tumorigenesis [317].

Importantly, structural features of the tumor influence anti-tumor immune functions, including tumor tissue infiltration, interactions with other cellular components, and contact with target tumor cells [111,318]. Moreover, ECM stiffness is a significant marker of cancer progression and invasion, and it is associated with immune escape [113,319,320]. Increased tumor stiffness creates physical obstacles to immune cell infiltration [318,321,322].

Tumors can evade immune cell infiltration by inducing desmoplastic reactions. These occur when CAFs, stromal cells, and ECM all stick together, forming dense connective tissue enveloping tumor nests, creating a desmoplastic stroma. This dense stroma impairs both immune cell access to and contact with malignant cells. Therefore, desmoplastic reactions not only correlate with reduced immune infiltrates but also with poor prognosis and increased drug resistance [323,324,325,326].

Beyond the direct organization of cellular and non-cellular components in desmoplastic structures, the specific configuration of the ECM also profoundly impacts immune cell trafficking modalities in situ. Indeed, T cells preferentially navigate through areas where the matrix exhibits a loose organization, thus favoring random locomotion, which is more efficient for immune surveillance functions. However, because of the TME unconventional topography, appropriate interactions between adhesion molecules on the surface of immune cells and their receptors are obstructed. Likewise, chemokine and cytokine signals are dispersed and/or less attainable, thereby disorienting the immune cell locomotion. As a consequence, proper and effective immune cell mobilization and distribution within the TME is impaired, resulting in inefficient T-cell-mediated anti-tumor immunity [111,327,328,329].

Finally, the signaling pathway necessary for DC differentiation and antigen presentation functions are also affected. A study showed that ECM stiffness decreased the CCR7 expression on mature DCs, leading to altered chemotaxis, therefore impeding the contact between DCs and T cells. Moreover, C-type lectin expression was also reduced in immature DCs, resulting in differential antigen internalization and reduced cross-presentation, thus dampening the anti-tumor effector functions [330].

## 5. Conclusions

Immune cell infiltration within tumors and its prognostic value have recently taken center stage in the field of tumor immunology. Indeed, assessing the immune contexture within the TME has a crucial interest to cancer immunologists for evaluating the type and magnitude of anti-tumor immune responses. Furthermore, it may guide personalized therapies targeting specific features of the patient’s immune system, the TME, or both.

Immune cell infiltration within the tumor niche is a critical step toward achieving cancer eradication. As we have summarized in this review, adhesion cues and chemokine signaling are key both for regulating proper immune cell trafficking in the tumor niche and for establishing effective crosstalk in the TME, thus ultimately achieving anti-tumor cytotoxicity. Consequently, hampering adhesion-dependent immune cell functions allows cancers to escape immune control at multiple levels. In some instances, mutations of specific proteins may have a dual role by inducing both tumor cell transformation and alteration of immune cell infiltrates and anti-tumor responses. This is the case for the tumor suppressor and cell polarity regulator adenomatous polyposis coli, or Apc, which is mutated in virtually all cases of sporadic colorectal cancers and in an inherited form of malignant polyposis (familial adenomatous polyposis, FAP) [331]. T cells from FAP patients displayed discrete alterations in cell migration ex vivo [332,333] and defective VLA-4-dependent adhesion [332,333]. Moreover, defective killing and inefficient destruction of cancer spheroids resulted from a reduced engagement of effector T cells from Apc-mutant mice with tumor cells [334]. These defects are likely to reduce human T cells’ ability to infiltrate tumors and eliminate them, thus contributing to the cancer onset in FAP patients. Similarly, Y42 mutations in RHOA, drivers of gastric cancer (GC), have been correlated with the creation of an immunosuppressive TME. Indeed, RHOA mutations in GC cells led to the accumulation of free fatty acids in the TME, which in turn fostered the selective recruitment of Tregs [335]. Of note, some effects of current immune checkpoint-based therapies may be mediated by the modulation of T-cell migration, as it has been recently suggested in the case of PD-1-dependent signaling [336]. Hence, further dissection of these pathways may be a field of future investigations with the aim of improving the efficiency of current approaches.

Targeting tumor-mediated disruptions in immune cell adhesion and migration could be key to enhancing cancer therapies, such as CAR-T therapy. Despite the latter having presented successful treatment efficacy for hematological malignancies, this was limited in solid tumors. Indeed, one of the main challenges of CAR-T cell therapy is to allow these cells to migrate to the tumor and persist within the TME [337]. Thus, it will be interesting to assess the efficacy of combining CAR-T cells with strategies favoring their intratumoral recruitment and motility. Indeed, current immunotherapies are building strategies to boost the infiltration of effector immune cells into tumors and their interaction with malignant cells [141,142,338]. Some strategies aim to boost T cell metabolism to enhance their migration [339], while others focus on targeting external elements. One such strategy involves increasing the concentration of anti-tumor chemokines within the TME, thus fostering the recruitment of anti-tumor effector immune cells. For example, oncolytic viruses such as NG-641, which express CXCL9, CXCL10, and IFN-α, are currently undergoing clinical trials in patients with epithelial tumors [340]. Another strategy implies the engineering of therapeutic cells, such as CAR-T cells, to be “armed” with chemokine receptors, such as CXCR6, which more effectively drive their infiltration to the tumor site [341]. Finally, manipulation of the tumor stroma and its interaction with soluble regulators and/or immune effector cells may be an interesting area for future clinical development [342].

Meanwhile, advanced techniques allow the assessment of leukocyte dynamics in vivo, such as in mouse or zebrafish models, or under complex microenvironments, such as microfabricated channels or microfluidic chips [343,344,345,346,347,348], enabling the dissection of intrinsic cellular features and cell cross-talk within the niche under both physiological and pathological conditions. Those techniques can help improve our knowledge about the differential behavior of TME-infiltrating immune cells and integrate it with the characterization of adhesion and chemokine signaling distribution. Moreover, transcriptomic profiling of tumor transition states should consider TME chemokine and/or adhesion cues to predict the establishment of an immunosuppressive environment and to identify new prognostic or diagnostic markers. Finally, physical methods modulating T-cell position and motility are being tested in in vivo models of solid tumors. For example, an external magnetic field has been used to facilitate the precise positioning as well as to promote tumor recognition and killing by adoptively-transferred T cells previously engineered with dual-binding magnetic nanoparticles [349,350]. Thus, comparable approaches may bring significant advances in cancer immunology and inspire novel therapeutic opportunities.

It should be considered that, since adhesion molecules, chemokines, and their receptors are expressed by multiple tissues and cell types, targeting these elements may raise some concerns about specificity and may lead to off-target effects that need to be carefully evaluated during preclinical and clinical trials. Nevertheless, combining novel strategies targeting cell migration and adhesion with already established anti-tumor treatments, such as genetically engineered T cells or immune checkpoint inhibitors, holds great potential for improving cancer therapy and can pave the way for new avenues of intervention.

## Figures and Tables

**Table 1:** Summary of the clinical trials mentioned in the review.

Trial ID	Target	Combination Therapy	Phase	Tumor Disease	Ref.
**Tumor vasculature**					
NCT04191889	Apatinib (anti-VEGFR-2)	Camrelizumab (anti-PD-1)	II	Hepatocellular carcinoma	[168]
NCT04047017	Apatinib (anti-VEGFR-2)	Camrelizumab (anti-PD-1)	II	High-risk chemorefractory or relapsed gestational trophoblastic neoplasia	[169]
NCT03603756	Apatinib (anti-VEGFR-2)	Camrelizumab (anti-PD-1), Chemotherapy	II	Advanced esophageal squamous cell carcinoma	[170]
NCT03092895	Apatinib (anti-VEGFR-2)	Camrelizumab (anti-PD-1)	Ib/II	Advanced primary liver cancer	[171]
NCT02588170	Sulfatinib (anti-VEGFR-1/2/3)	no	III	Advanced neuroendocrine tumor	[172]
NCT02314819	Fruquintinib (anti-VEGFR-1/2/3)	no	III	Metastatic colorectal cancer	[173]
NCT02811861	Lenvatinib (anti-VEGFR-1/2/3)	Pembrolizumab (anti-PD-1)	III	Advanced renal carcinoma	[182]
NCT05281471	Bevacizumab (anti-VEGFR-1/2/3)	Chemotherapy	III	Platinum-resistant/refractory ovarian cancer	[183]
NCT03038100	Bevacizumab (anti-VEGFR-1/2/3)	Atezolizumab (anti-PD-L1), Chemotherapy	III	Stage III or IV Ovarian Cancer	[184]
**CXCR4-CXCL12 axis**					
NCT00903968	Plerixafor (anti-CXCR4)	Chemotherapy	I/II	Multiple myeloma	[186]
NCT02826486	Motixafortide (anti-CXCR4)	Chemotherapy	II	Pancreatic cancer	[187]
NCT01837095	Balixafortide (anti-CXCR4)	Chemotherapy	I	HER2-negative metastatic breast cancer	[188]
NCT01439568	LY2510924 (anti-CXCR4)	Chemotherapy	II	Extensive-disease small cell lung cancer	[189]
NCT01391130	LY2510924 (anti-CXCR4)	Sunitinib (anti-multiple receptor tyrosine kinases)	II	Metastatic Renal Cell Carcinoma	[190]

## Data Availability

The study did not report any new results or data.

## Abbreviations

APC	Antigen-presenting cell
Apc	Adenomatous polyposis coli
BCG	Bacillus Calmette-Guerin
BM	Basement membrane
CAF	Cancer-associated fibroblasts
CAM	Cell adhesion molecule
CAR-T	Chimeric antigen receptor T cell
CD	Cluster of differentiation
CLEVER-1	Common lymphatic endothelial and vascular endothelial receptor 1
CTL	Cytotoxic T cell
DC	Dendritic cell
EC	Endothelial cell
ECM	Extracellular matrix
EpCAM	Epithelial cell adhesion molecule
ET_B_R	Endothelin B receptor
FDA	Food and Drug Administration
FGF	Fibroblast growth factor
GC	Gastric cancer
GPCR	G-protein-coupled receptor
HCC	Hepatocellular carcinoma
HIF	Hypoxia-inducible factor
ICAM	Intercellular adhesion molecule
IL	Interleukin
IS	Immunological synapse
JAM-A	Junctional adhesion molecule A
LFA-1	Lymphocyte function-associated antigen-1
M1/2	Macrophage type-1/type-2
MMP	Metalloprotease
MDSC	Myeloid-derived suppressor cell
MHC	Major histocompatibility complex
NK	Natural killer
NO	Nitric oxide
NOS	NO synthase
PD-1	Programmed cell death protein 1
PDA	Pancreatic ductal adenocarcinoma
PD-L1	Programmed death-ligand 1
PDGF	Platelet-derived growth factor
PKA1	Protein kinase A1
PSGL-1	P-selectin glycoprotein ligand-1
PTEN	Phosphatase and tensin homolog
ROS	Reactive oxygen species
SI-CLP	Stabilin-1-interacting chitinase-like protein
TAM	Tumor-associated macrophage
TAN	Tumor-associated neutrophil
TCR	T cell receptor
Th	T helper
TME	Tumor microenvironment
TLS	Tertiary lymphoid structure
Treg	T regulatory
VAP-1	Vascular-associated protein-1
VCAM	Vascular cell adhesion molecule
VEGF	Vasculature endothelial growth factor
VEGFR	VEGF receptor
VLA-4	Very-late antigen-4
ZO-1	Zonula occludens-1
